# A novel TXNIP-based mechanism for Cx43-mediated regulation of oxidative drug injury

**DOI:** 10.1111/jcmm.12641

**Published:** 2015-07-08

**Authors:** Kun Gao, Yuan Chi, Xiling Zhang, Hui Zhang, Gang Li, Wei Sun, Masayuki Takeda, Jian Yao

**Affiliations:** a Department of Molecular Signaling, Interdisciplinary Graduate School of Medicine and Engineering, University of YamanashiChuo, Yamanashi, Japan; b Department of Nephrology, Affiliated Hospital of Nanjing University of Chinese MedicineNanjing, Jiangsu, China; c Department of Urology, Interdisciplinary Graduate School of Medicine and Engineering, University of YamanashiChuo, Yamanashi, Japan; d Department of Urology, Liaoning Cancer Hospital & InstituteShenyang, Liaoning, China

**Keywords:** connexin 43, gap junction, oxidative stress, cytotoxicity, TXNIP, Akt

## Abstract

Gap junctions (GJs) play an important role in the regulation of cell response to many drugs. However, little is known about their mechanisms. Using an *in vitro* model of cytotoxicity induced by geneticin (G418), we explored the potential signalling mechanisms involved. Incubation of cells with G418 resulted in cell death, as indicated by the change in cell morphology, loss of cell viability and activation of caspase-3. Before the onset of cell injury, G418 induced reactive oxygen species (ROS) generation, activated oxidative sensitive kinase P38 and caused a shift of connexin 43 (Cx43) from non-phosphorylated form to hyperphosphorylated form. These changes were largely prevented by antioxidants, suggesting an implication of oxidative stress. Downregulation of Cx43 with inhibitors or siRNA suppressed the expression of thioredoxin-interacting protein (TXNIP), activated Akt and protected cells against the toxicity of G418. Further analysis revealed that inhibition of TXNIP with siRNA activated Akt and reproduced the protective effect of Cx43-inhibiting agents, whereas suppression of Akt sensitized cells to the toxicity of G418. Furthermore, interference of TXNIP/Akt also affected puromycin- and adriamycin-induced cell injury. Our study thus characterized TXNIP as a presently unrecognized molecule implicated in the regulatory actions of Cx43 on oxidative drug injury. Targeting Cx43/TXNIP/Akt signalling cascade might be a promising approach to modulate cell response to drugs.

## Introduction

Gap junctions (GJs), formed by the specific protein termed connexin (Cx), are integral membrane organelles that allow the direct cytoplasmic exchange of ions and low-molecular weight metabolites between adjacent cells. One GJ is composed of two hemichannels, each is provided by one of two neighbouring cells. Up to date, more than 20 different isoforms of Cx proteins have been identified. Among them, Cx43 is most extensively investigated because of its ubiquitous expression in almost all cell types. Cells in normal tissues are linked by GJs, which play an important role in the maintenance of tissue integrity and homeostasis 1–3.

Gap junctions regulate many cellular processes. Altered GJs and its forming protein Cx have been reported in various pathological situations and recognized as important pathogenic factors contributing to the initiation and development of certain diseases 1–5. One of the well-documented actions of GJs is to regulate cell response to cytotoxic agents 6–11. In tumour cells, increased GJs and/or its forming protein Cxs sensitize cell to tumour chemotherapy, whereas inhibition of GJs/Cxs render tumour cell resistance to the cytotoxicity of cancer-killing drugs 12–16. Manipulation of GJs has been considered as an effective approach to overcome drug resistance and enhance the efficacy of chemotherapy 15–18. Beside tumour cells, GJs also regulate drug responses in normal cells, including renal and hepatic cells 7,10,11. Different from tumour cells, drug cytotoxicity in these cells is the leading cause of renal and hepatic impairment. It is also the major reason for limitation or even interruption of the use of many drugs in the clinic 19–21. Modification of GJs under these situations might help prevent or alleviate drug-induced cell injury. To develop novel and effective strategies to modulate cell responses to drugs, it is highly desirable to understand the regulatory mechanisms.

The mechanisms by which GJs regulate cell reactivity to drugs are still poorly understood. Given that GJs are conductive channels that mediate the synchronous behaviour of coupled cells in response to various stimulants, the effect of GJs on drug-induced cell injury has long been considered to be through transmission and propagation of ‘death signals’ among the cells 22. Indeed, GJ-mediated transmission of calcium, reactive oxygen species (ROS) and cytochrome C has been reported to propagate cell death 23–25. Recently, however, there are also evidence indicating an existence of GJ communication-independent actions 26,27. Connexins interact with many structural and functional proteins. Altered expression of Cxs itself could lead to activation of several important signalling molecules, such as Src and NF-κb 28,29. These molecules mediate some of the biological actions of Cxs 15,28–32.

The cytotoxicity and non-cytotoxic epigenetic effects of many drugs is mediated by oxidative stress 33. The implication of increased ROS generation and activated ROS-sensitive kinases in drug-elicited cell death, as well as their prevention by antioxidants has been well documented 11,34–38. It is likely that some of the actions of GJs on drug response might be through regulation of oxidative stress. In fact, GJs have been shown to play an important role in many oxidative stress-associated pathological situations 9,11,33,34,39. At present, our knowledge about the molecules regulated by GJs and pivotally involved in regulation of oxidative stress is still limited. The purpose of this study is to identify the potential molecules and signalling mechanisms involved in the regulation of oxidative drug injury by GJs/Cxs. Here, we present our result showing that thioredoxin-interacting protein (TXNIP) and Akt are downstream targets of Cx43, participating in the regulation of drug-induced oxidative cell injury.

## Material and methods

### Reagents

DMEM/F12 was purchased from Nacalai Tesque, Inc (Tokyo, Japan). RPMI 1640, foetal bovine serum (FBS), trypsin/ethylenediaminetetraacetic acid, H_2_O_2_, glycyrrhizin (GZA), glutathione (GSH) were obtained from Wako Pure Chemical (Osaka, Japan). Anti-Cx43 antibodies, 18α-glycyrrhetinic acid (α-GA), 18β-GA, lindane, flufenamic acid (FFA), carbenoxolone (CA), *N*-Acetyl-l-Cysteine (NAC), geneticin (G418) were purchased from Sigma-Aldrich (Tokyo, Japan). Antibodies against phospho-p38 MAPK (Thr180/Tyr182), β-tubulin, caspase-3 and horseradish peroxidase-conjugated anti-rabbit or mouse IgG were obtained from Cell Signaling Inc (Beverly, MA, USA). Antibody against TXNIP was purchased from MBL International (Woburn, MA, USA).

### Cells

Tubular proximal epithelial cell line, normal rat kidney (NRK)-E52 (from the American Type Culture Collection, Rockville, MD, USA), was maintained in DMEM/F12 supplemented with 100 U/ml penicillin G, 100 mg/ml streptomycin, 0.25 mg/ml amphotericin B and 5% FBS. Murine podocytes were kindly provided by Dr. Karlhans Englich (University of Heidelberg, Heidelberg, Germany) and cultured with RPMI-1640 medium supplemented with 5% FBS. Mouse foetal fibroblasts were derived from the foetal offspring of mating pairs of heterozygous Cx43 knockout mice (B6, 129-Gja1 < tm1Kdr+/− mice; Jackson Laboratories, Bar Harbor, ME, USA), using a method described by Ehrlich *et al*. with minor modifications 8,40. Briefly, paired mouse forelimbs were taken from foetuses at day 18 of gestation, minced, and digested in DMEM/F12 containing 0.1% collagenase for 30 min. Freed cells were collected and cultured in DMEM/F12 medium containing 15% FBS. Cells at passages between 5 and 15 were used for this study. Genotypes of individual mice and established cell lines were analysed by PCR. All animal experimental procedures were approved by the Animal Experimental Committee of Yamanashi University. Mice were housed in containment facilities of the Animal Center and maintained on a regular 12:12-hr light/dark cycle with food and water.

### Assessment of cell viability with CCK-8 reagent

Cells were seeded into 96-well cultured plate and allowed to grow for 72 hrs in DMEM/F-12 medium containing 0.5% FBS. After that, they were exposed to various stimuli in the presence or absence of the specified chemicals for the indicated times. Cell Counting Kit-8 (CCK-8) reagent (Dojindo, Kumamoto, Japan) was added to each well and incubated for addition 0.5–3 hrs before measurement of optical density (OD) with a spectrometer at the wave of 450 nm. Cell viability was expressed as percentage of control cells.

### Detection of superoxide anion and ROS production

The generation of superoxide anion (O_2_^•−^) and ROS was detected by using a commercially available kit from Enzo (Tokyo, Japan) following the manual of the manufacturer, as previously described 11,38. Briefly, cells in 96-well plates were pre-incubated with O_2_^•−^ detection reagent (orange) or oxidative stress detection reagent (green) for 1 hr and exposed to various stimulants for the indicated times. The immunofluorescent image was visualized and captured using an Olympus IX71 inverted fluorescence microscope (Olympus, Hachioji-shi, Tokyo, Japan) equipped with a standard red and green fluorescence cube.

### Transient transfection of cells with siRNA

NRK cells were transiently transfected with siRNA specifically targeting Cx43 or TXNIP (Qiagen, Tokyo, Japan), or a negative control siRNA (All Stars Negative Control siRNA) at a final concentration of 20 nM using Hyperfect transfection reagent for 48 hrs. After that, cells were either left untreated or exposed to various stimuli for the indicated times. Cellular protein was extracted and subjected to Western blot analysis for Cx43 and TXNIP. Cell viability was evaluated by CCK-8 assay.

### Western blot analysis

Total cellular protein was extracted by suspending the cells in SDS lysis buffer (62.5 mM Tris-HCl, 2% SDS, 10% glycerol) together with freshly added proteinase inhibitor cocktail (Nacalai Tesque, Kyoto, Japan). Lysates were incubated on ice for 15 min. with intermittent mixing and then centrifuged at 5000 × g for 5 min. at 4°C. Supernatant was recovered and protein concentration was determined using the Micro BCA Protein Assay Kit (Pierce, Rockford, IL, USA). Western blot was performed by the enhanced chemiluminescence system. Briefly, extracted cellular proteins were separated by 10% SDS-polyacrylamide gels and electrotransferred onto polyvinylidine difluoride membranes. After blocking with 3% bovine albumin or 5% non-fat dry milk in PBS, the membranes were incubated with primary antibody. After washing, the membranes were probed with horseradish peroxidase-conjugated anti-rabbit or antimouse IgG, and the bands were visualized by the enhanced chemiluminescence system (Amersham Biosciences, Buckinghamshire, UK). The chemiluminescent signal is captured with a Fujifilm luminescent image LAS-1000 analyser (Fujifilm, Tokyo, Japan) and quantified with densitometric software Fujifilm Image Gauge. To confirm the equal protein loading, western blot for β-tubulin were performed. Quantitative measurements of the bands were done using ImageJ software.

### Statistical analysis

Values are expressed as mean ± SD or SE. Comparison of two populations was made by Student’s *t*-test. For multiple comparisons, one-way anova followed by Dunnett’s test was employed. Both analyses were done by using the SigmaStat statistical software (Jandel Scientific, San Rafael, CA, USA). *P* < 0.05 was considered to be a statistically significant difference.

## Results

### Aminoglycoside induces oxidative cell injury

**Figure 1:**
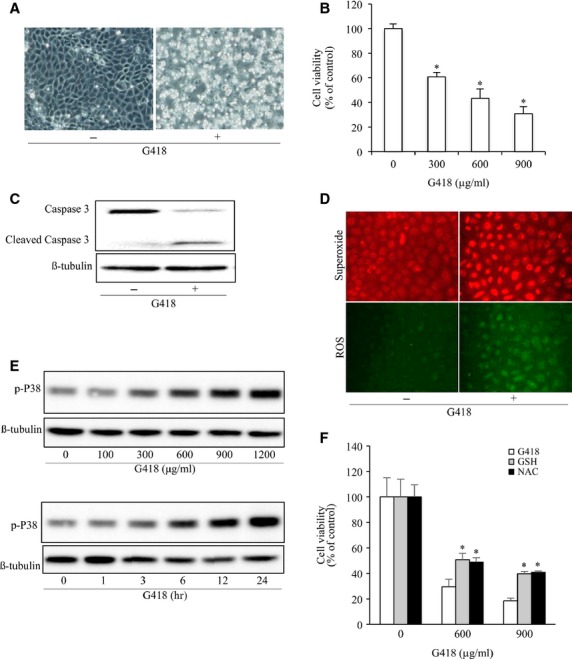
Aminoglycoside elicits oxidative cell injury. (A) Induction of cell shape change by G418. NRK cells were exposed to 600 μg/ml G418 for 48 hrs. Cell morphology was photographed using phase-contrast microscopy (magnification, ×100). (B) Effect of G418 on cell viability. NRK cells were exposed to the indicated concentrations of G418 for 48 hrs. The cell viability was evaluated by CCK-8 assay. Data are expressed as percentage of living cells against the untreated control (mean ± SD, *n* = 4). *P < 0.05 *versus* untreated control. (C) Activation of caspase-3 by G418. NRK cells were exposed to 600 μg/ml G418 for 48 hrs and subjected to Western blot analysis of caspase-3. The top band represents procaspase-3 (M.W. 35,000) and the bottom band indicates its cleaved, mature form (M.W. 17,000). (D) Effects of G418 on O_2_^•−^ and ROS production. Cells were loaded with O_2_^•−^ and ROS detection reagent for 1 hr and stimulated with 900 μg/ml G418 for 24 hrs. After that, they were subjected to fluorescent microscopy (magnification, ×400). (E) Induction of P38 phosphorylation by G418. Cells were incubated with the indicated concentrations of G418 for 12 hrs or 600 μg/ml G418 for the indicated intervals. Cellular lysates were subjected to Western blot analysis for phosphorylated P38. (F) Effect of antioxidants on cell viability. Cells were exposed to the indicated concentrations of G418 for 48 hrs in the presence or absence of 5 mM GSH and 10 mM NAC. The cell viability was evaluated by CCK-8 assay. Data are expressed as percentage of living cells against the untreated control (mean ± SD, *n* = 4; *P < 0.05).

G418 is an aminoglycoside antibiotic similar to gentamicin in both structure and function, which is widely used in selection of genetically engineered cells 41. Because it is much more potent than gentamicin in cytotoxicity, we have used it to investigate the mechanisms involved in drug-induced renal cell injury. Exposure of rat renal tubular epithelial cell line NRK to G418 led to cell injury, as demonstrated by the appearance of round-shaped, loosely attached cells (Fig. 1A) and loss of cell viability (Fig. 1B). Western blot analysis of caspase-3 revealed that G418 treatment resulted in activation of caspase-3, as indicated by the appearance of an additional cleaved, low-molecular band (Fig. 1C). These observations thus indicate that G418 induces apoptotic cell death in renal tubular cells.

Given that oxidative stress is one of the major mechanisms that mediate the toxicity of many drugs, including aminoglycoside 42,43, we therefore examined its role in G418-induced cell injury. Using fluorescent probers, we detected an elevation in ROS and O_2_^•−^ in G418-treated cells, as demonstrated by the markedly increased fluorescent intensity (Fig. 1D). Consistently, G418 also activated oxidative stress-sensitive kinase P38 (Fig. 1E). To determine the role of oxidative stress in G418-induced cell injury, we examined cell viability in the presence of antioxidant GSH and NAC. Figure 1F shows that both agents significantly attenuated the cytotoxicity of G418, indicating an involvement of oxidative stress.

### Cx43 regulates cell reactivity to G418

**Figure 2:**
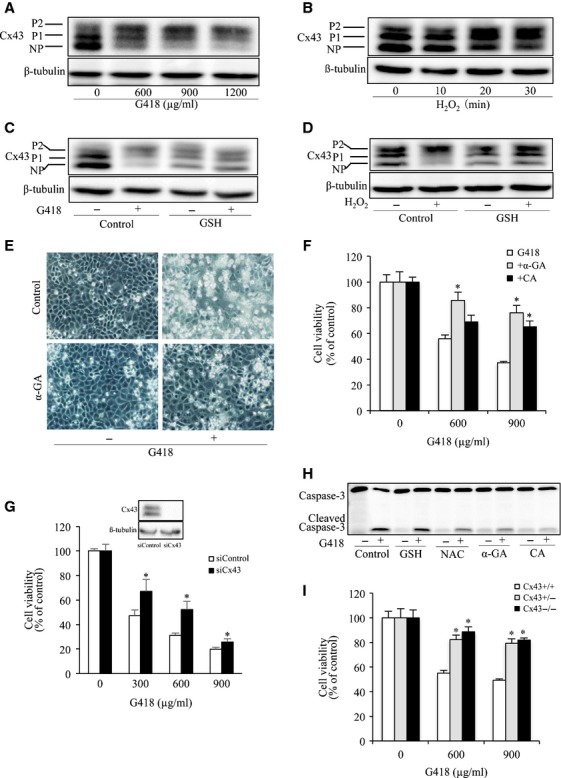
GJs contributed to aminoglycoside-induced NRK-E52 cell injury. (A and B) Influence of G418 and H_2_O_2_ on Cx43 expression and phosphorylation. NRK cells were exposed to the indicated concentrations of G418 for 24 hrs (A) or H_2_O_2_ for the indicated time intervals (B). Cellular proteins were subjected to Western blot analysis of Cx43 and loading control β-tubulin. NP and P denote non-phosphorylated and phosphorylated Cx43 respectively. (C and D) Effects of GSH on G418- or H_2_O_2_-induced Cx43 expression and phosphorylation. Cells were exposing to 600 μg/ml G418 or 50 μM H_2_O_2_ in the presence or absence of 5 mM GSH for 24 hrs (C) and 30 min. respectively. Cellular lysates were subjected to Western blot analysis for Cx43. (E) Effects of GJ inhibitor on cell morphology. Cells were exposed to 600 μg/ml G418 with or without 7.5 μM α-GA for 48 hrs. Cell morphology was photographed using phase-contrast microscopy (magnification, ×100). (F) Effects of GJ inhibitors on cell viability. NRK-E52 cells were exposed to the indicated concentrations of G418 in the presence or absence of 7.5 μM α-GA or 10 μM CA for 48 hrs. The cell viability was evaluated by CCK-8 assay. Data are expressed as percentage of living cells against the untreated control (mean ± SE, *n* = 4; *P < 0.05). (G) Effect of Cx43 siRNA on G418-induced cell injury. Cells transfected with Cx43 siRNA or control siRNA were exposed to the indicated concentrations of G418 for 72 hrs. Then the cellular viability was evaluated through CCK-8 assay. Data are expressed as percentage of living cells, compared with the siRNA control (mean ± SD, *n* = 4; *P < 0.05 *versus* siRNA control). (H) Effects of antioxidants and GJ inhibitors on G418-induced activation of caspase-3. Cells were pre-treated with 5 mM GSH, 10 mM NAC, 7.5 μM α-GA or 10 μM CA for 1 hr before exposing to 600 μg/ml G418 for an additional 24 hrs. Cellular lysates were subjected to Western blot analysis for caspase-3. The top band represents procaspase-3 (M.W. 35,000) and the bottom band indicates its cleaved, mature form (M.W. 17,000). (I) Effects of G418 on cell viability in foetal fibroblast cells. C43+/+, Cx43+/− and Cx43−/− fibroblasts were incubated with indicated concentrations of G418 for 24 hrs. The cell viability was evaluated by CCK-8 assay. Data are expressed as percentage of living cells against the untreated control (mean ± SD, *n* = 4; *P < 0.05 *versus* G418 alone).

Connexin 43 is reported to regulate cell susceptibility to several cytotoxic drugs 7–11. To confirm the effects under the current experimental settings, we first examined the influence of G418 on Cx43 expression. In Western blots, Cx43 protein was detected as three bands (NP, P1, P2), corresponding to the non-phosphorylated (NP), phosphorylated (P1) and hyperphosphorylated form (P2) respectively. G418 stimulation resulted in a shift of Cx43 from non-phosphorylated (P0) to hyper-phosphorylated Cx43 (P2) (Fig. 2A). The shift was blunted by supplement of cells with antioxidant GSH and mimicked by H_2_O_2_ 44, indicative of a mediating role of oxidative stress (Fig. 2B–D).

We then proceeded to examine the role of Cx43 in cell injury. In consistent with our previous report 7, inhibition of GJs with chemical inhibitor α-GA or CA, or downregulation of Cx43 with siRNA attenuated G418-induced cell injury in NRK cells, as indicated by improved cell morphology, increased cell viability and reduced activation of caspase-3 (Fig. 2E–H). Furthermore, fibroblasts derived from Cx43 heterozygous (Cx43+/−) and knockout (Cx43−/−) mouse were more resistant to the cytotoxicity of G418, as compared with those from wild-type littermates (Cx43+/+) (Fig. 2I). Collectively, these results indicate that Cx43 regulates cell sensitivity to G418 45.

### TXNIP contributes to Cx43-mediated regulation of drug response

**Figure 3:**
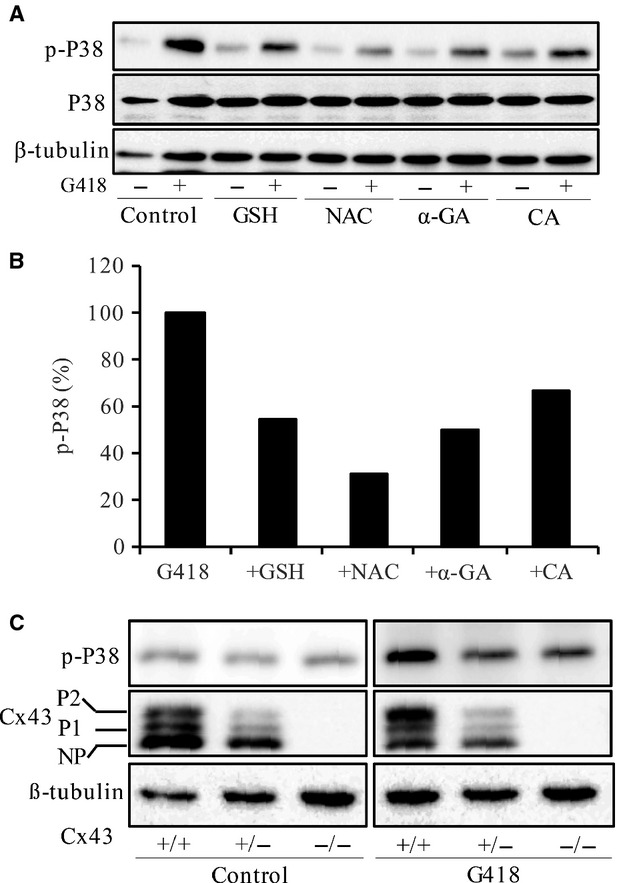
Cx43 regulates aminoglycoside-induced activation of P38. (A and B) Effects of antioxidants and GJ inhibitors on G418-induced activation of P38. Cells were incubated with 5 mM GSH, 10 mM NAC, 7.5 μM α-GA and 10 μM CA for 1 hr before exposing to 600 μg/ml G418 for an additional of 24 hrs. Cellular lysates were subjected to Western blot analysis for total P38 and phosphorylated P38. The percentage of phosphorylated P38 in G418-treated cells with or without GJ inhibitors is shown in the lower panel of B. Quantitative measurement of the band density was performed with ImageJ 1.46 software. The phosphorylated levels of P38 were normalized to total p38, and are expressed as percentage of phosphorylation relative to G418-treated control. Note the obviously reduced level of phosphorylated P38 in antioxidant- or GJ inhibitor-treated cells. (C) Induction of P38 and Cx43 by G418 in foetal fibroblast cells. Cx43+/+, Cx43+/− and Cx43−/− foetal fibroblast cells were treated with or without 600 μg/ml G418 for 12 hrs. Then cellular lysates were subjected to Western blot analysis for P38 and Cx43. Note the different abundance of Cx43 and shift of Cx43 to phosphorylated form in Cx43+/+ and Cx43+/− cells.

Because oxidative stress is involved in the cytotoxicity of aminoglycosides 43, we therefore examined the potential influence of altered Cx43 on intracellular oxidative status. For this purpose, we examined the phosphorylated level of P38, an oxidative stress-sensitive kinase. Figure 3A and B show that P38 activation induced by G418 was attenuated by antioxidant GSH and NAC. It was also attenuated by GJ inhibitor α-GA and CA. Consistently, Cx43−/− cells displayed a weak activation of P38 in response to G418 in comparison with Cx43+/+ fibroblasts (Fig. 3C). Furthermore, G418-induced shift of Cx43 from non-phosphorylated form to hyperphosphorylated one was more pronounced in Cx43+/+ cells than that in Cx43+/− cells. These results indicate that Cx43 might influence oxidative stress induced by G418.

**Figure 4:**
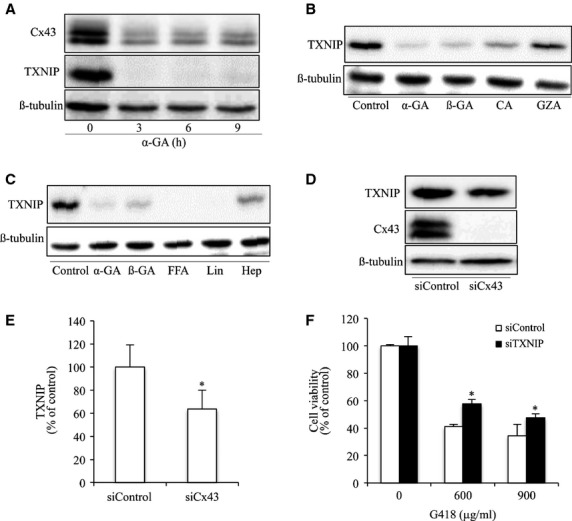
Inhibition of GJ suppresses TXNIP. (A) Effects of GJ inhibitors on TXNIP. NRK cells were incubated with 7.5 μM α-GA for the indicated time intervals. Cellular lysates were subjected to Western blot analysis for TXNIP and Cx43. (B and C) Effects of structural analogue of α-GA and different GJ inhibitors on TXNIP expression. NRK cells were incubated with 7.5 μM α-GA, 7.5 μM ß-GA, 10 μM CA, 15 μM GZA (B), 50 μM FFA, 100 μM lindane (Lin) and 2 mM heptanol (Hep) (C) for 6 hrs. Cellular lysates were subjected to Western blot analysis for TXNIP. (D and E) Downregulation of Cx43 with siRNA on TXNIP expression. NRK cells were transfected with either Cx43 siRNA or control siRNA for 48 hrs. Cellular lysates were subjected to Western blot analysis for TXNIP and Cx43. Equal loading of protein per lane was verified by probing the blots with an anti-β-tubulin antibody. Results are representatives of 3 separate experiments. Densitometric analyses of TXNIP in D was done by using ImageJ software and are expressed as percentage of the control (mean ± SE, *n* = 3; *P < 0.05 compared with the siRNA control). (F) Effect of TXNIP siRNA on G418-induced cell injury. NRK cells were transfected with either TXNIP siRNA or control siRNA for 48 hrs. The cellular viability was evaluated through CCK-8 assay. Data are expressed as percentage of living cells, compared with the siRNA control (mean ± SD, *n* = 4; *P < 0.05 *versus* siRNA control).

Given that TXNIP is implicated in oxidative cell injury induced by several cytotoxic drugs 38,46,47*,* we therefore tested the possible involvement of TXNIP. To this end, we examined the influence of Cx43 on TXNIP protein levels. As shown in Figure 4A, treatment of cells with GJ inhibitor α-GA caused a rapid decrease in Cx43 level, which was associated with a marked reduction in TXNIP. This effect was mimicked by β-GA and CA, two structural analogues of α-GA that disrupt GJs 48, but not by GZA that does not affect GJs 49 (Fig. 4B). The similar effect was achieved by the structurally different GJ inhibitors, FFA and lindane. Another GJ inhibitor heptanol, which suppresses GJ intercellular communication without great influence on Cx protein level 50,51, to a lesser extent, also affected TXNIP expression (Fig. 4C). Consistent with the result obtained from chemical inhibitors, downregulation of Cx43 with siRNA also reduced the basal level of TXNIP (Fig. 4D and E). These results indicate that downregulation of Cx43 suppresses TXNIP level. We then proceeded to investigate the role of TXNIP in G418-induced oxidative cell injury. Figure 4F shows that downregulation of TXNIP with siRNA significantly increased cell resistance to the cytotoxicity of G418. These data indicate that TXNIP is critically involved in the regulatory effect of Cx43 on cell susceptibility to G418.

### Akt is implicated in Cx43/TXNIP- mediated regulation of drug response

**Figure 5:**
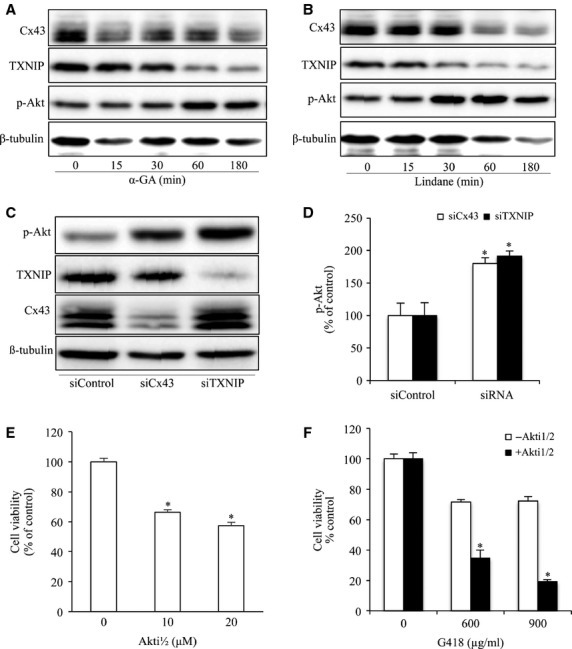
Inhibition of Cx43 and TXNIP activate AKT. (A and B) Effects of GJ inhibitors on Akt phosphorylation and TXNIP. NRK cells were treated with 7.5 μM α-GA or 100 μM lindane for the indicated time intervals. Cellular lysate were subjected to Western blot analysis of phosphorylated Akt, TXNIP and Cx43. (C and D) Downregulation of Cx43 or TXNIP on Akt phosphorylation. NRK cells were transfected with either Cx43 siRNA or TXNIP siRNA for 48 hrs. Cellular lysates were subjected to Western blot analysis for phosphorylated Akt, TXNIP and Cx43. Equal loading of protein per lane was verified by probing the blots with an anti-β-tubulin antibody. Densitometric analyses of phosphorylated Akt were done by using ImageJ software and are expressed as percentage of the control (mean ± SE, *n* = 3; *P < 0.05 compared with the siRNA control). (E) Effects of Akt inhibitor on cell viability. NRK cells were incubated with the indicated various concentrations of Akt inhibitor Akti1/2 for 60 hrs. The cell viability was evaluated by CCK-8 assay. Data are expressed as percentage of living cells against the untreated control (mean ± SD, *n* = 4; *P < 0.05 *versus* untreated control). (F) Effects of Akt inhibitor on G418-induced cell injury. NRKs were exposed to the indicated concentrations of G418 for 18 hrs in the presence or absence of Akt inhibitor, 10 μM Akti1/2. The cell viability was evaluated by CCK-8 assay. Data are expressed as percentage of living cells against the untreated control (mean ± SD, *n* = 4; *P < 0.05 *versus* G418 alone).

Our previous study characterized Akt as a potential mechanism underlying the protective effect of GJ inhibitors on the cytotoxicity of G418 7. Given that TXNIP has been recently reported to be able to regulate Akt 52, we therefore examined the possible implication of TXNIP. Figure 5A and B show that two structurally different GJ inhibitors, α-GA and lindane, suppressed TXNIP expression, which was followed by an elevation in AKT phosphorylation. Downregulation of Cx43 with siRNA also led to a reduction in TXNIP and an activation of Akt. Further analysis using TXNIP siRNA revealed that downregulation of TXNIP also activated Akt (Fig. 5C and D). Collectively, these results indicated that inhibition of Cx43 suppressed TXNIP, which in turn activated Akt. To confirm the role of Akt in cell injury, we examined cell viability in the presence of Akt inhibitor Akti1/2. Figure 4D and F show that Akti1/2 caused a loss of cell viability and exaggerated the toxicity of G418.

### Cx43/TXNIP/Akt signalling cascade regulates cell responses to several different cytotoxic drugs

**Figure 6:**
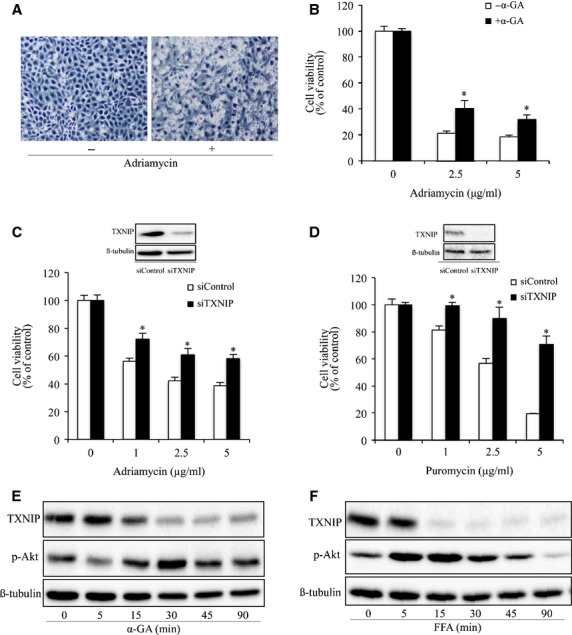
Cx43/TXNIP/Akt signalling cascade regulates cell response to puromycin and adriamycin. (A) Induction of NRK-E52 cell shape change by adriamycin. NRK-E52 cells were exposed to adriamycin (1 μg/ml) for 24 hrs. Cell morphology was photographed using phase-contrast microscopy (magnification, ×100). (B) Effect of GJ inhibitor on adriamycin-induced loss of cell viability. NRK cells were exposed to the indicated concentrations of adriamycin in the presence or absence of 7.5 μM α-GA for 20 hrs. The cell viability was evaluated by CCK-8 assay. Data are expressed as percentage of living cells against the untreated control (mean ± SD, *n* = 4). *P < 0.05 *versus* adriamycin alone. (C) Effects of TXNIP siRNA on adriamycin-induced cell injury. NRK-E52 cells were transfected with either TXNIP siRNA or control siRNA for 24 hrs. The transfected cells were incubated with the indicated concentrations of adriamycin for 30 hrs. The cellular viability was evaluated through CCK-8 assay. Data are expressed as percentage of living cells, compared with the siRNA control (mean ± SD, *n* = 4; *P < 0.05 *versus* siRNA control). (D) Effects of TXNIP siRNA on puromycin-induced cell injury. Cells were transfected with either TXNIP siRNA or control siRNA for 24 hrs. The transfected cells were incubated with indicated various concentrations of puromycin for 30 hrs. The cellular viability was evaluated through CCK-8 assay. Data are expressed as percentage of living cells, compared with the siRNA control (mean ± SE, *n* = 4; *P < 0.05 *versus* siRNA control). (E) Effects of GJ inhibitors on Akt phosphorylation and TXNIP in podocytes. Podocytes were treated with 7.5 μM α-GA or 50 μM FFA for the indicated time intervals. Cellular lysate were subjected to Western blot analysis of phosphorylated Akt and TXNIP. Equal loading of protein per lane was verified by reprobing the blots with an anti-β-tubulin antibody.

To determine whether the regulatory effect of Cx43 and TXNIP on cell response to cytotoxic drug is stimulant-specific, we examined their roles in adriamycin- and puromycin-induced cell injury in NRK. As shown in Figure 6A–C, the cytotoxicity of adriamycin to NRK cells was significantly inhibited by treatment of cells with GJ inhibitor α-GA or siRNA against TXNIP. Downregulation of TXNIP with siRNA also significantly protected cells against the toxicity of puromycin.

We have described that that GJ inhibitors protected communication-deficient podocytes from puromycin-induced oxidative cell injury. However, little information is available regarding their mechanisms. Here, we tested whether Cx43 also regulate TXNIP/AKT in communication-deficient cells 11. Incubation of podocytes with GJ inhibitor also resulted in a reduction in TXNIP and an increase in Akt phosphorylation (Fig. 6D–F). These results thus indicate that modulation of TXNIP by Cx43 could be through its communication-independent actions and that regulation of TXNIP/Akt cascade could be an important mechanism by which Cx43 regulates cell response to oxidative cell injury.

## Discussion

In this study, we demonstrated that Cx43 regulated cytotoxic cell response to drug-induced oxidative injury. Furthermore, we characterized TXNIP/Akt as downstream targets of Cx43 contributing to the altered cell responses. Our study thus unravels a novel mechanism by which Cx43 regulates oxidative cell injury and opens a new window towards our further understanding of the roles and mechanisms of Cx43 in the regulation of cell behaviours.

Accumulating evidence indicates that GJs affect cell sensitivity to cytotoxic drugs 7–11,53,54. Most of the reports have shown that decreased GJs increases cell resistance, whereas increased GJs sensitize cells to the cytotoxic effects of drugs 7–11,15,16,55. This property of GJs has been observed in many types of cells and in cell response to a wide range of cytotoxic agents, including acetaminophen, aminoglycosides, MG132, cadmium, cisplatin, vinblastine and glutamate 7–11,15,49,56. Consistent with these previous observations, we demonstrated that inhibition of Cx43 with chemical inhibitors or siRNA significantly prevented the cytotoxicity of aminoglycoside, puromycin and adriamycin on renal cells. Our results thus support the notion that GJs are important factors controlling cell sensitivity to drugs.

The cytotoxicity of many drugs is mediated by oxidative stress 36. It also held true for aminoglycoside G418 used in the current investigation. The toxicity of G418 was associated with an elevated ROS and was greatly attenuated in the presence of antioxidants. In our previous reports, we have described that the toxicity of puromycin and adriamycin on renal cells was also mediated by oxidative stress 11,38. In this context, it is conceivable that the effect of Cx43 on drug sensitivity could be attributed to its actions on intracellular oxidative status. Consistent with this notion, Cx43 has been implicated in many pathological situations associated with oxidative stress 9,11. For examples, Cx43 regulates cell survival under hypoxic and ischaemic situations 57,58. It also determines cell fate to cadmium, a heavy metal that induces cell death through depletion of intracellular GSH 9. It appears that Cx43 regulates cell response to oxidative stress caused by multiple insults.

The question naturally occurs as to how GJs regulated intracellular oxidative status? Most of the previous studies have shown that GJs regulate the intracellular redox status through channel-mediated transmission of signalling molecules, like superoxide and calcium ions. These molecules have been described to be able to pass through GJs to propagate toxic responses in several different pathological situations 23,59,60. In addition, apoptotic signals, such as cytochrome C, can directly pass through GJs 25. GJs could also regulate intracellular oxidative status through hemichannel-mediated intra- and extracellular exchange of small molecules that are critical for redox homeostasis, such as GSH and Nicotinamide Adenine Dinucleotide Phosphate Hydrogen (NADPH) 9,61. We have reported that hemichannels- mediated efflux of GSH impacted intracellular redox status and enhanced cell susceptibility to cadmium-elicited cell injury 9. Beside these channel-associated regulatory mechanisms, Cx43 also exerts channel-independent actions. We have observed that downregulation of Cx43 similarly attenuated puromycin-induced cell injury in communication-deficient podocytes 11. An early study by Huang *et al*. showed that the communication- independent downregulation of antiapoptotic Bcl2 might contribute to the enhanced cell susceptibility to chemotherapeutic drugs in glioblastoma tumour cells transfected with a wild-type Cx43 62. At present, the molecular mechanisms behind the channel-independent actions of Cx43 on drug response are still unclear. It could be related to Cx C-terminal mediated interaction with intracellular signalling molecules, such as src. Studies by Sato and Takano, *et al*. have demonstrated that knockdown of Cx32 enhanced tumour cell resistance to vinblastine through src-induced expression of multidrug resistance gene-1 15,63.

In this study, we demonstrated, for the first time, a critical implication of TXNIP in the regulatory effects of Cx43. This is shown by the observations: (*i*) inhibition of Cx43 with several structurally and functionally different chemical inhibitors or downregulation of Cx43 with siRNA similarly suppressed TXNIP expression; and (*ii*) reduced TXNIP enhanced cell resistance to the toxic drugs. As an important redox regulator, TXNIP suppresses anti-oxidative activities of thioredoxin through binding to its active cysteine residue 64. It has been reported that overexpression of TXNIP increases ROS generation and oxidative cell death 65, whereas suppression of TXNIP enhances cells resistance to oxidative cell injury 47,65. Furthermore, implication of TXNIP in drug-induced oxidative injury has also been documented. TXNIP mediated cyclosporine- and glucose-induced renal cell apoptotic injury 46,47.

The mechanism by which TXNIP regulates oxidative stress could be related to its action on thioredoxin, one of the major cellular defence mechanisms against oxidative stress 47,64. In our previous study, we have demonstrated that thioredoxin regulated cell response to adriamycin. Suppression of thioredoxin with chemical inhibitor Px-12 or siRNA sensitized renal cell to adriamycin 38. In this investigation, the reduced expression of TXNIP might lead to an enhanced activity of thioredoxin, thus increasing cell resistance to oxidative cell injury. Apart from its action on thioredoxin, TXNIP might also affect cell behaviours through regulation of Akt. Downregulation of TXNIP activated Akt. The effect might be related to the reported inhibitory effect of TXNIP on PTEN, an upstream regulator and phosphatase of Akt 66. The implication of Akt in protection of cells against various stresses has been extensively documented 67,68. Inhibition of Akt in this study potentiated the toxicity of G418. These observations thus indicate that Cx43 might regulate drug response through its downstream signalling molecules, TXNIP and Akt.

At present, the mechanism by which Cx43 regulated TXNIP expression is unclear. It appeared that this effect of Cx43 was most likely to be communication-independent. This notion is supported by the observation that Cx43-inhibiting chemicals also suppressed TXNIP in podocytes. In our previous report, we have shown that podocytes used in our investigation expressed Cx43 protein, but did not form functional intercellular communication because of lack of Cx43 protein at cell-to-cell contact region 11. However, we cannot completely exclude the participation of channel-dependent mechanisms, because heptanol that inhibits channel ability without interference of Cx43 protein levels, to a lesser extent, also suppressed TXNIP expression. The details of the mechanism involved will be the subject of future studies.

Our findings could have significant implications. First, we revealed an unreported mechanism by which Cx43 regulates cellular oxidative status and cell survival after exposure to cytotoxic inducing chemicals. TXNIP and Akt have multifaceted biological functions 65,67. They regulate redox signalling, metabolism, inflammation and cancer. Regulation of TXNIP/Akt by Cx43 highlights the importance of Cx43 in these pathological situations. Second, our study indicates that targeting Cx43/TXNIP/Akt could be developed to enhance or attenuate the toxicity of certain cytotoxic drugs. Depending on pathological contexts, it might be used to potentiate the tumour-killing efficacy of chemotherapy or to protect the cells from the undesirable side effects of drugs. Collectively, we identified TXNIP/Akt as downstream targets of Cx43, which mediated the regulatory actions of Cx43 on cell response to cytotoxic chemicals, and probably other biological functions as well.

## References

[b1] Saez JC, Berthoud VM, Branes MC (2003). Plasma membrane channels formed by connexins: their regulation and functions. Physiol Rev.

[b2] Yao J, Oite T, Kitamura M (2009). Gap junctional intercellular communication in the juxtaglomerular apparatus. Am J Physiol Renal Physiol.

[b3] Nielsen MS, Axelsen LN, Sorgen PL (2012). Gap junctions. Compr Physiol.

[b4] Loewenstein WR, Kanno Y (1966). Intercellular communication and the control of tissue growth: lack of communication between cancer cells. Nature.

[b5] Yotti LP, Chang CC, Trosko JE (1979). Elimination of metabolic cooperation in Chinese hamster cells by a tumor promoter. Science.

[b6] Wilson MR, Close TW, Trosko JE (2000). Cell population dynamics (apoptosis, mitosis, and cell-cell communication) during disruption of homeostasis. Exp Cell Res.

[b7] Yao JA, Huang T, Fang X (2010). Disruption of gap junctions attenuates aminoglycoside-elicited renal tubular cell injury. Br J Pharmacol.

[b8] Huang T, Zhu Y, Fang X (2010). Gap junctions sensitize cancer cells to proteasome inhibitor MG132-induced apoptosis. Cancer Sci.

[b9] Fang X, Huang T, Zhu Y (2011). Connexin43 hemichannels contribute to cadmium-induced oxidative stress and cell injury. Antioxid Redox Signal.

[b10] Patel SJ, Milwid JM, King KR (2012). Gap junction inhibition prevents drug-induced liver toxicity and fulminant hepatic failure. Nat Biotechnol.

[b11] Yan QJ, Gao K, Chi Y (2012). NADPH oxidase-mediated upregulation of connexin43 contributes to podocyte injury. Free Radic Biol Med.

[b12] Trosko JE, Ruch RJ (2002). Gap junctions as targets for cancer chemoprevention and chemotherapy. Curr Drug Targets.

[b13] Chipman JK, Mally A, Edwards GO (2003). Disruption of gap junctions in toxicity and carcinogenicity. Toxicol Sci.

[b14] Ogawa T, Hayashi T, Tokunou M (2005). Suberoylanilide hydroxamic acid enhances gap junctional intercellular communication via acetylation of histone containing connexin 43 gene locus. Cancer Res.

[b15] Sato H, Senba H, Virgona N (2007). Connexi n 32 potentiates vinblastine-induced cytotoxicity in renal cell carcinoma cells. Mol Carcinog.

[b16] Kandouz M, Batist G (2010). Gap junctions and connexins as therapeutic targets in cancer. Expert Opin Ther Targets.

[b17] Trosko JE, Ruch RJ (1998). Cell-cell communication in carcinogenesis. Front Biosci.

[b18] Salameh A, Dhein S (2005). Pharmacology of gap junctions. New pharmacological targets for treatment of arrhythmia, seizure and cancer?. Biochim Biophys Acta.

[b19] Choudhury D, Ahmed Z (2006). Drug-associated renal dysfunction and injury. Nat Clin Prac Nephrol.

[b20] Lee WM (2003). Medical progress: drug-induced hepatotoxicity. N Engl J Med.

[b21] Smith CR, Lipsky JJ, Laskin OL (1980). Double-blind comparison of the nephrotoxicity and auditory toxicity of gentamicin and tobramycin. N Engl J Med.

[b22] Krysko DV, Leybaert L, Vandenabeele P (2005). Gap junctions and the propagation of cell survival and cell death signals. Apoptosis.

[b23] Lin JHC, Weigel H, Cotrina ML (1998). Gap-junction-mediated propagation and amplification of cell injury. Nat Neurosci.

[b24] Feine I, Pinkas I, Salomon Y (2012). Local oxidative stress expansion through endothelial cells - a key role for gap junction intercellular communication. PLoS ONE.

[b25] Cusato K, Ripps H, Zakevicius J (2006). Gap junctions remain open during cytochrome c-induced cell death: relationship of conductance to ‘bystander’ cell killing. Cell Death Differ.

[b26] Zhang YW, Kaneda M, Morita I (2003). The gap junction-independent tumor-suppressing effect of connexin 43. J Biol Chem.

[b27] Zhou JZ, Jiang JX (2014). Gap junction and hemichannel-independent actions of connexins on cell and tissue functions–an update. FEBS Lett.

[b28] Gangoso E, Ezan P, Valle-Casuso JC (2012). Reduced connexin43 expression correlates with c-Src activation, proliferation, and glucose uptake in reactive astrocytes after an excitotoxic insult. Glia.

[b29] Xie X, Lan T, Chang X (2013). Connexin43 mediates NF-kappaB signalling activation induced by high glucose in GMCs: involvement of c-Src. Cell Commun Signal.

[b30] Toyofuku T, Akamatsu Y, Zhang H (2001). c-Src regulates the interaction between connexin-43 and ZO-1 in cardiac myocytes. J Biol Chem.

[b31] Lin R, Warn-Cramer BJ, Kurata WE (2001). v-Src phosphorylation of connexin 43 on Tyr247 and Tyr265 disrupts gap junctional communication. J Cell Biol.

[b32] Alonso F, Krattinger N, Mazzolai L (2010). An angiotensin II- and NF-kappaB-dependent mechanism increases connexin 43 in murine arteries targeted by renin-dependent hypertension. Cardiovasc Res.

[b33] Upham BL, Trosko JE (2009). Oxidative-dependent integration of signal transduction with intercellular gap junctional communication in the control of gene expression. Antioxid Redox Signal.

[b34] Ruch RJ, Cheng SJ, Klaunig JE (1989). Prevention of cytotoxicity and inhibition of intercellular communication by antioxidant catechins isolated from Chinese green tea. Carcinogenesis.

[b35] Upham BL, Guzvic M, Scott J (2007). Inhibition of gap junctional intercellular communication and activation of mitogen-activated protein kinase by tumor-promoting organic peroxides and protection by resveratrol. Nutr Cancer.

[b36] Pereira CV, Nadanaciva S, Oliveira PJ (2012). The contribution of oxidative stress to drug-induced organ toxicity and its detection *in vitro* and *in vivo*. Expert Opin Drug Metabo Toxicol.

[b37] Leone A, Longo C, Trosko JE (2012). The chemopreventive role of dietary phytochemicals through gap junctional intercellular communication. Phytochem Rev.

[b38] Gao K, Chi Y, Sun W (2014). 5′-AMP-activated protein kinase attenuates adriamycin-induced oxidative podocyte injury through thioredoxin-mediated suppression of the apoptosis signal-regulating kinase 1-P38 signaling pathway. Mol Pharmacol.

[b39] Thompson RJ, Zhou N, MacVicar BA (2006). Ischemia opens neuronal gap junction hemichannels. Science.

[b40] Ehrlich HP, Gabbiani G, Meda P (2000). Cell coupling modulates the contraction of fibroblast-populated collagen lattices. J Cell Physiol.

[b41] Davies J, Jimenez A (1980). A new selective agent for eukaryotic cloning vectors. Am J Trop Med Hyg.

[b42] Davis W, Ronai Z, Tew KD (2001). Cellular thiols and reactive oxygen species in drug-induced apoptosis. J Pharmacol Exp Ther.

[b43] Lopez-Novoa JM, Quiros Y, Vicente L (2011). New insights into the mechanism of aminoglycoside nephrotoxicity: an integrative point of view. Kidney Int.

[b44] Upham BL, Kang KS, Cho HY (1997). Hydrogen peroxide inhibits gap junctional intercellular communication in glutathione sufficient but not glutathione deficient cells. Carcinogenesis.

[b45] Sheridan JD, DeMello WC (1987). Cell communication and growth. Cell to cell communication.

[b46] O’connell S, Tuite N, Slattery C (2012). Cyclosporine A-induced oxidative stress in human renal mesangial cells: a role for ERK 1/2 MAPK signaling. Toxicol Sci.

[b47] Schulze PC, Yoshioka J, Takahashi T (2004). Hyperglycemia promotes oxidative stress through inhibition of thioredoxin function by thioredoxin-interacting protein. J Biol Chem.

[b48] Juszczak GR, Swiergiel AH (2009). Properties of gap junction blockers and their behavioural, cognitive and electrophysiological effects: animal and human studies. Prog Neuropsychopharmacol Biol Psychiatry.

[b49] Ozog MA, Siushansian R, Naus CCG (2002). Blocked gap junctional coupling increases glutamate-induced neurotoxicity in neuron-astrocyte co-cultures. J Neuropathol Exp Neurol.

[b50] Takenskwak BR, Jongsma HJ, Rook MB (1992). Mechanism of heptanol-induced uncoupling of cardiac gap-junctions - a perforated patch-clamp study. Am J Physiol.

[b51] Cronier L, Frendo JL, Defamie N (2003). Requirement of cap junctional intercellular communication for human villous trophoblast differentiation. Biology Reprod.

[b52] Chen JQ, Hui ST, Couto FM (2008). Thioredoxin-interacting protein deficiency induces Akt/Bcl-xL signaling and pancreatic beta-cell mass and protects against diabetes. FASEB J.

[b53] Rivedal E, Witz G, Leithe E (2010). Gap junction intercellular communication and benzene toxicity. Chem Biol Interact.

[b54] Lee J, Yim YS, Ko SJ (2011). Gap junctions contribute to astrocytic resistance against zinc toxicity. Brain Res Bull.

[b55] Peterson-Roth E, Brdlik CM, Glazer PM (2009). Src-induced cisplatin resistance mediated by cell-to-cell communication. Cancer Res.

[b56] Wang YW, Wang Q, Zhang SZ (2014). Baicalein increases the cytotoxicity of cisplatin by enhancing gap junction intercellular communication. Mol Med Rep.

[b57] Lin JHC, Lou N, Kang N (2008). A central role of connexin 43 in hypoxic preconditioning. J Neurosci.

[b58] Wang N, De Vuyst E, Ponsaerts R (2013). Selective inhibition of Cx43 hemichannels by Gap19 and its impact on myocardial ischemia/reperfusion injury. Basic Res Cardiol.

[b59] Azzam EI, de Toledo SM, Little JB (2001). Direct evidence for the participation of gap junction-mediated intercellular communication in the transmission of damage signals from alpha-particle irradiated to nonirradiated cells. Proc Natl Acad Sci USA.

[b60] Krutovskikh VA, Piccoli C, Yamasaki H (2002). Gap junction intercellular communication propagates cell death in cancerous cells. Oncogene.

[b61] Bruzzone S, Guida L, Zocchi E (2001). Connexin 43 hemi channels mediate Ca^2+^-regulated transmembrane NAD(+) fluxes in intact cells. FASEB J.

[b62] Huang RP, Hossain MZ, Huang R (2001). Connexin 43 (cx43) enhances chemotherapy-induced apoptosis in human glioblastoma cells. Int J Cancer.

[b63] Takano Y, Iwata H, Yano Y (2010). Up-regulation of connexin 32 gene by 5-aza-2′-deoxycytidine enhances vinblastine-induced cytotoxicity in human renal carcinoma cells via the activation of JNK signalling. Biochem Pharmacol.

[b64] Nishiyama A, Matsui M, Iwata S (1999). Identification of thioredoxin-binding protein-2/vitamin D-3 up-regulated protein 1 as a negative regulator of thioredoxin function and expression. J Biol Chem.

[b65] Zhou JB, Chng WJ (2013). Roles of thioredoxin binding protein (TXNIP) in oxidative stress, apoptosis and cancer. Mitochondrion.

[b66] Hui STY, Andres AM, Miller AK (2008). Txnip balances metabolic and growth signaling via PTEN disulfide reduction. Proc Natl Acad Sci USA.

[b67] Hers I, Vincent EE, Tavare JM (2011). Akt signalling in health and disease. Cell Signal.

[b68] Ikeyama S, Kokkonen G, Shack S (2001). Loss in oxidative stress tolerance with aging linked to reduced extracellular signal-regulated kinase and Akt kinase activities. FASEB J.

